# Supplementary material for: Influence of Chinese Herbal Formula on Bone Characteristics of Cobb Broiler Chickens
Source: Genes (Basel). 2022 Oct 15;13(10):1865. doi: 10.3390/genes13101865 (PMC9601401; doi:10.3390/genes13101865)
Supplement: Supplementary file 1 [file genes-13-01865-s001.zip › genes-1961569-supplementary.pdf]

Table S1. Primer name, sequence, product size and annealing temperature.

Name	Sequence	Annealing temperature(℃)	Product size (bp)
β-actin	F: tggactcctacaaccaacgg<br>R: catcctccttgaactcgcag	59.7	258
BMP-2	F: gtttgtggtggaggtggttc<br>R: gtccacatacaacggatgcc	56.0	238
OPG	F: ttcctactcgttccacaccc<br>R: tgctccttgttttgctgctt	57.1	200
VDR	F: cgatgttcacctgtccgttc<br>R: cgatgactttctgctgctcc	59.0	231
Runx2	F: cttgagccagatgacttcgc<br>R: gacgggcggtgtataggtaa	56.8	229
RANK	F: agaaatcttgccagcgttcc<br>R: tttctggggaggtgaaagca	59.0	186
RANKL	F: agagctgtgcagaaggaagt<br>R: ggtttgcctggcctttgtta	59.0	214
TNF-α	F: atgaaccctccgcagtactc<br>R: aagaggccaccacacgaca	60.0	200
